# Upcycling of Cr-Containing Sulfate Waste into Efficient FeCrO_3_/Fe_2_O_3_ Catalysts for CO_2_ Hydrogenation Reaction

**DOI:** 10.3390/ma17071598

**Published:** 2024-03-31

**Authors:** Yongqi Liu, Shasha Chu, Yuebing Xu, Xinyu Chen, Hao Zhou, Jinlin Li, Yanjie Ren, Xintai Su

**Affiliations:** 1 Guangdong Provincial Key Laboratory of Solid Wastes Pollution Control and Recycling, School of Environment and Energy, South China University of Technology, Guangzhou 510006, China; 2 School of Chemical and Material Engineering, Jiangnan University, Wuxi 214122, China; 3 Xinjiang Qinghua Energy Group Co., Ltd., Yining County 835100, China

**Keywords:** Cr-containing sodium sulfate, FeCrO_3_/Fe_2_O_3_ catalyst, CO_2_ hydrogenation reaction, detoxification

## Abstract

Upcycling Cr-containing sulfate waste into catalysts for CO_2_ hydrogenation reaction benefits both pollution mitigation and economic sustainability. In this study, FeCrO_3_/Fe_2_O_3_ catalysts were successfully prepared by a simple hydrothermal method using Cr-containing sodium sulfate (Cr-SS) as a Cr source for efficient conversion and stable treatment of Cr. The removal rate of Cr in Cr-SS can reach 99.9% at the optimized hydrothermal conditions. When the synthesized catalysts were activated and used for the CO_2_ hydrogenation reaction, a 50% increase in CO_2_ conversion was achieved compared with the catalyst prepared by impregnation with a comparable amount of Cr. According to the extraction and risk assessment code (RAC) of the Reference Office of the European Community Bureau (BCR), the synthesized FeCrO_3_/Fe_2_O_3_ is risk-free. This work not only realizes the detoxification of the Cr-SS but transfers Cr into stable FeCrO_3_ for application in a catalytic field, which provides a strategy for the harmless disposal and resource utilization of Cr-containing hazardous waste.

## 1. Introduction

Cr-containing sodium sulfate (Cr-SS) is a by-product in the production process of the chrome salt industry. Cr (VI) in Cr-SS has the characteristics of high mobility, high toxicity, and carcinogenesis [1,2]. If it is discharged into the environment without proper treatment, it will pose a serious threat to the ecological environment and human health. In fact, Cr has been designated as hazardous waste in the China National Hazardous Waste List (2016), making the safe treatment and resource utilization of Cr-SS a pressing issue within chromium-related industries.

Currently, treatment methods for Cr-containing waste are categorized into harmless [3] and extraction methods [4]. Commonly employed techniques include wet reduction [5], high-temperature roasting [6], bioremediation [7,8,9], immobilization/stabilization [10,11], and physico-chemical extraction methods [4,12,13,14]. Among these methods, wet reduction and physico-chemical extraction are commonly used disposal measures. Wet reduction involves the use of acids or bases to transfer Cr (VI) from the solid phase to the liquid phase and then chemically reduce it to Cr (III), which is less toxic. Although this method is simple, it usually produces acid–base waste liquid and consumes a large amount of reducing agent [15]. The physico-chemical extraction method achieves sufficient extraction of Cr by controlling the phase transition of solid particles [16]. Although this method realizes the effective extraction of chromium, it has not been fully explored in the utilization of resources. Considering that Cr is a strategic metal resource, the annual waste of Cr resources due to inefficient practices is enormous [17,18]. Therefore, it is urgent to develop an efficient, stable, and economical method to recover chromium from Cr-containing sodium sulfate.

We propose the idea of detoxifying Cr from Cr-containing sodium sulfate (Cr-SS) and selectively converting it into functional materials to achieve a balance between environmental and economic considerations. Since Cr exists mainly in the form of Fe-Cr crystals in the natural environment [19], the conversion of Cr into stable Fe-Cr crystals can significantly reduce its ecotoxicity due to oxidation. On the other hand, iron–chromium oxide (FeCrO_3_) has a similar molecular formula to ilmenite, which display excellent chemical stability, thermal stability, magnetic properties, and specific catalytic capabilities [20,21,22]. Therefore, if the Cr in Cr-SS can be selectively converted into FeCrO_3_ by direct reaction with iron, the simultaneous detoxification and stabilization of Cr can be effectively achieved, which provides a new strategy for the resource utilization of Cr-containing wastes. Some studies have mentioned the hydrothermal conversion of Cr(VI) to stable ferrite. Lan [23] synthesized FeCr_2_O_4_ by hydrothermal treatment of chromium-containing wastewater, while Xie [24] applied hydrothermal treatment to chromium sludge and synthesized FeCr_2_O_4_ for advanced persulfate oxidation. However, there has been no research on the conversion of chromium from Cr-containing waste to FeCrO_3_ for high-value utilization.

In this study, Cr (VI) detoxification and the preparation of FeCrO_3_/Fe_2_O_3_ catalytic material were realized by a hydrothermal process using Cr-SS as raw material. The obtained material is activated and used for a carbon dioxide hydrogenation reaction. The research objectives include the following: (1) investigating the effects of different hydrothermal treatment conditions on the fixation and stabilization of Cr. (2) The prepared FeCrO_3_/Fe_2_O_3_, Cr/Fe_2_O_3_, and Fe_2_O_3_ were analyzed utilizing analytical characterization methods. (3) Elucidating the mechanism of Cr(VI) conversion to FeCrO_3_. (4) Conducting CO_2_ hydrogenation experiments on FeCrO_3_/Fe_2_O_3_, Cr/Fe_2_O_3_, and Fe_2_O_3_ to investigate differences in catalytic performance. (5) Determining the speciation of Cr in different Fe-Cr materials using the European Community Bureau of Reference (BCR) method and assessing their environmental risks using the risk assessment code (RAC) method. This study provides a convenient method for the efficient detoxification of Cr-SS and fixation of Cr elements, and also offers a feasible strategy for the effective disposal and resource utilization of Cr-containing solid waste.

## 2. Experimental

### 2.1. Materials

The Cr-SS was obtained from Chongqing Minfeng Chemical Co., Ltd. (Chongqing, China). It is a by-product of the process used to prepare chromate and dichromate from chrome ore using the alkali method. The collected samples were dried at 80 °C, crushed, and sieved to ensure uniform compositions. After ICP determination, the total Cr content in Cr-SS is 1.03 mg/g. The chemical reagents used in this study included anhydrous sodium sulfite (Na_2_SO_3_, AR, 99%), ferric sulfate (Fe_2_(SO_4_)_3_, AR, 99%), sodium sulfate (Na_2_SO_4_, AR, 99%), and urea (CO(NH_2_)_2_, AR, 99%) purchased from Aladdin Reagent Co., Ltd. (Shanghai, China). Deionized water was used in all the experiments. All chemicals were used as received and without further purification. Unless otherwise noted, all samples tested herein were screened through a 200 mesh (0.074 mm) sieve.

### 2.2. Experimental

#### 2.2.1. Preparation of FeCrO_3_/FeOOH and FeCrO_3_/Fe_2_O_3_

Firstly, 1.0 g of Cr-SS was dissolved in 20 mL of deionized water, then Cr(VI) was utterly reduced to Cr(III) by adding Na_2_SO_3_. Simultaneously, Fe_2_(SO_4_)_3_ and CO(NH_2_)_2_ were introduced to form a homogeneous solution, transferred into a 100 mL Teflon-lined stainless autoclave, and maintained at 180 °C for 12 h. After air-cooling to room temperature, the precipitate was filtered and washed with deionized water and anhydrous alcohol several times and then dried in an oven at 80 °C for 10 h. The dried product is denoted as FeCrO_3_/FeOOH. Finally, the FeCrO_3_/Fe_2_O_3_ was obtained by calcinating the FeCrO_3_/FeOOH powder in air at 500 °C for 2 h with a 10 °C/min heating rate (the process of calcination at 500 °C for 2 h was regarded as high-temperature activation).

The solution was collected after hydrothermal reaction, and then dried to obtain the sodium salt after removing Cr (Cr-SS-AR). After ICP testing, the Cr content in Cr-SS-AR is 3.18 × 10^−4^ mg/g. The Cr removal rate of the method can be calculated to be 99.9% based on Formula (1).

Cr removal rate = (c(Cr-SS) − c(Cr-SS-AR))/c(Cr-SS) × 100%(1)

#### 2.2.2. Preparation of Fe_2_O_3_ and Cr/Fe_2_O_3_

To compare the catalytic performance of FeCrO_3_/Fe_2_O_3_ material in CO_2_ hydrogenation synthesis, a control sample was prepared using Na_2_SO_4_ instead of Cr-SS. The following steps were followed to obtain Fe_2_O_3_ and Cr/Fe_2_O_3_ materials.

Cr-SS was replaced with Na_2_SO_4_ under the same experimental conditions as FeCrO_3_/Fe_2_O_3_. The final product obtained is Fe_2_O_3_.

An isovolumetric impregnation method was employed, using the previously obtained Fe_2_O_3_ as the impregnation cocatalyst. The impregnated cocatalyst was dried in an oven at 120 °C for 2 h, and 1.0 g was taken for the determination of water absorption. Based on the water absorption of Fe_2_O_3_, Na_2_CrO_4_ was used as the Cr source, and deionized water was used as the solvent to formulate the impregnation solution with the corresponding concentration, so as to ensure that the final Cr loading of the catalyst was 0.3%. A total of 1.0 g of dried sample was put into an eggplant-shaped bottle, adding the calculated amount of impregnation solution, impregnated at room temperature for 1 h, stirring every 10 min to ensure full impregnation, then vacuum drying at the end of impregnation for 12 h to obtain a red powder, and the obtained red powder was put into a muffle furnace, and the temperature was raised to 500 °C at a rate of 10 °C/min and kept for 2 h, to obtain the final catalyst product, named as Cr/Fe_2_O_3_.

### 2.3. Characterization

The total metal concentrations were analyzed using an inductively coupled plasma emission spectrometer (ICP-OES, AvioTM200, PerkinElmer, Waltham, MA, USA). The Cr(VI) concentrations were quantified at 540 nm using a UV–vis spectrophotometer (UV2600, Shimadzu, Kyoto, Japan) based on the diphenylcarbazide colorimetric method (GB7467 [25] and GB/T15555.4-1995 [26]). The mineral phase compositions of the samples were determined using an X-ray diffractometer (XRD, D8Advance, Bruker, Saarbrücken, Germany) in continuous scanning mode with Cu-Kα radiation (λ = 1.5418 Å, 40 kV, 40 mA). The samples’ microstructure, morphology, and elemental analysis were examined using a scanning electron microscope equipped with an energy-dispersive X-ray spectrometer (SEM-EDS, Sigma300, Zeiss, Oberkochen, UK). The microstructure of the samples was observed using a transmission electron microscope (TEM, FEI Talos F200X, Waltham, MA, USA) in STEM mode at 200 kV. The valence changes of heavy metals on the sample surface were analyzed using X-ray photoelectron spectroscopy (XPS, Escalab 250Xi, Thermo Fisher Scientific, Waltham, MA, USA).

### 2.4. CO_2_ Hydrogenation Test

Catalytic performance evaluation of the synthesized catalyst for CO_2_/H_2_ methanation synthesis was conducted in a fixed-bed reactor. A 0.3 g sample of fresh catalyst and a 2.0 g sample of quartz sand were evenly blended and loaded into the reaction tube. The reaction was conducted under constant H_2_ flow rate conditions (100 mL/min). The temperature was increased at a rate of 2 °C/min until reaching 400 °C for a reduction period of 4 h. Subsequently, the temperature was lowered to 240 °C for evaluation under atmospheric pressure. The feed gas composition was V(H_2_): V(CO_2_) = 3:1 with a 100 mL/min flow rate. The Agilent (Santa Clara, CA, USA) GC-7820A gas chromatograph equipped with a thermal conductivity detector (TCD) and a flame ionization detector (FID) was used to analyze the online feed gas and gas products. The TCD, equipped with a Porapak Q column and an MS 5A packed column, was used to analyze components such as CO_2_, CO, CH_4_, etc. The FID, equipped with an Rt-Q-Bond column, was used to analyze hydrocarbon products ranging from C_2_ to C_7_. CO_2_ conversion ($X_{CO_{2}}\;$, %) and product selectivity (*S*, %) were calculated [27] as shown in Equations (2)–(5):

(2) $$X_{CO_{2}}=\frac{A_{CO_{2}in}-A_{CO_{2}out}}{A_{CO_{2}in}}\times 100\%$$

(3) $$S_{CO}=\frac{A_{CO\;out}\;}{A_{CO\;in}-A_{CO\;out\;}}\times 100\%$$

(4) $$S_{CH_{4}}=\frac{A_{CH_{4}out}\;}{A_{CH_{4}\;in}-A_{CH_{4}out}}\times 100\%$$

(5) $$S_{C_{2}-C_{7}}=100\%\;-S_{CH_{4}}-S_{CO}$$

In the equations, $X_{CO_{2}}\;$ represents the conversion rate of CO_2_. *S_CO_*, $S_{CH_{4}}$, and $S_{C_{2}-C_{7}}$ represent the selectivity of each component. $A_{CO_{2}}$, *A_CO_*, and $A_{CH_{4}}$ represent the molar concentration of each component, and the subscripts in and out represent the feed gas and the outlet gas, respectively.

### 2.5. Stability Test

Different forms of Cr in FeCrO_3_/Fe_2_O_3_ and Cr/Fe_2_O_3_ were extracted by a modified BCR four-step extraction method [28]. A sample weighing 0.8 g was mixed with 32 mL of a 0.1 mol/L acetic acid solution. The mixture was shaken for 16 h and then centrifuged to obtain extract 1 (exchangeable fraction F1) and residue 1. In residue 1, 32 mL of a 0.5 mol/L hydroxylamine hydrochloride solution was added, followed by shaking for 16 h. The mixture was centrifuged to obtain extract 2 (reducible fraction F2) and residue 2. For residue 2, 8 mL of an 8.8 mol/L hydrogen peroxide solution was added, and the mixture was heated until the volume was reduced to 1 mL. Then, 25 mL of a 1 mol/L ammonium acetate solution was added, followed by shaking for 16 h. The mixture was centrifuged to obtain extract 3 (oxidizable fraction F3) and residue 3. The dried sample from residue 3 was placed in a digestion bottle and mixed with 8 mL of aquaregia. The mixture was allowed to digest at room temperature for 5 h and then diluted to obtain extract 4 (residual fraction F4).

Formula (6) can be used to calculate the proportion of metal instability in the total content of the material, so as to assess the environmental risk level after the material is discarded [29].

RAC = F1 fraction/Total amount of heavy metals(6)

## 3. Results and Discussion

### 3.1. Detoxification and Targeted Transfer of Cr in Cr-SS

We determined the effect of conditions on Cr removal from Cr-SS by controlling the hydrothermal temperature, hydrothermal time, and the amounts of CO(NH_2_)_2_ and Fe_2_(SO_4_)_3_; the detailed information can be found in the ).

As shown in Figure S1, when the mass ratio of Cr-SS, Fe_2_(SO_4_)_3_, and CO(NH_2_)_2_ is 1 g:1 g:0.6 g, and the hydrothermal conditions are 180 °C and 12 h, the best Cr removal effect can be obtained. The XRD pattern of Cr-SS (Figure S2a) shows that the main phase is Na_2_SO_4_, and no crystalline compound of Cr was found. However, as shown in Figure S2b, Cr-SS is a yellow granular substance. This indicates that in Cr-SS, the content of Cr is relatively low, or Cr exists as amorphous Na_2_CrO_4_. After processing, the XRD pattern of Cr-SS-AR (Figure S2c) shows that the main phases of Cr-SS are Na_2_SO_4_ and (NH_4_)_2_SO_4_. The optical image of Cr-SS-AR (Figure S2d) shows the appearance of white particle crystals, indicating that Cr in Cr-SS has been removed. The Cr content in Cr-SS decreased from 41.2 mg/L to 1.27 × 10^−2^ mg/L before and after hydrothermal treatment, and no Fe content was detected (Table S1). In conclusion, the removal rate of Cr is 99.9% when the method is used for detoxification of Cr-SS, which is well-matched to the similar literature (see Table S2 for details on what the literature compares) [23,24,30,31,32].

**Figure 1:**
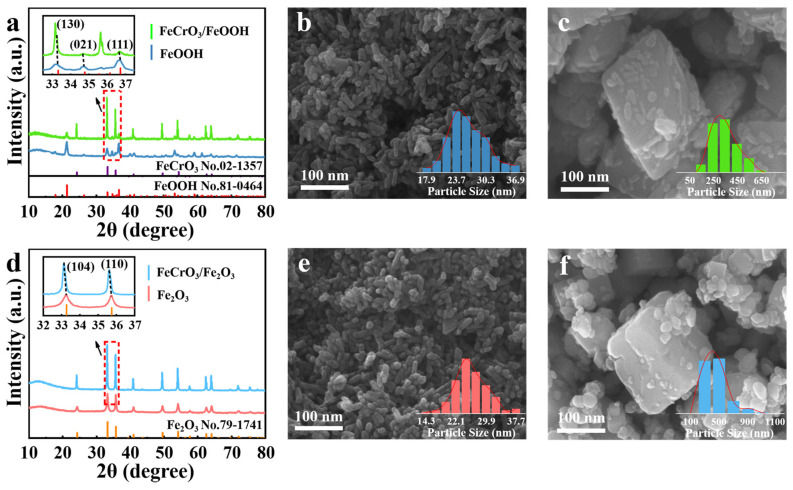
XRD patterns of (**a**) FeOOH and FeCrO_3_/FeOOH before high-temperature activation and (**d**) Fe_2_O_3_ and FeCrO_3_/Fe_2_O_3_ after being activated at 500 °C. SEM images of (**b**) FeOOH, (**c**) FeCrO_3_/FeOOH, (**e**) Fe_2_O_3_, and (**f**) FeCrO_3_/Fe_2_O_3_.

The cocatalysts were prepared using Cr-SS and Na_2_SO_4_ under the above optimum dechromiumization conditions and the XRD patterns and SEM images are shown in Figure 1. As shown in Figure 1, the characteristic peak of the material prepared by Na_2_SO_4_ corresponded to FeOOH, and the material was named FeOOH, and a new characteristic peak corresponding to FeCrO_3_ appeared in the material prepared by Cr-SS, and the material was named FeCrO_3_/FeOOH. By comparing the XRD spectra of the two materials, it was found that the peak corresponding to FeOOH in FeCrO_3_/FeOOH was shifted to a lower angle. According to the Bragg equation (2dsinθ = nλ), doping with heteroatoms of larger atomic radii increases the lattice constant, which leads to a shift in the diffraction peaks of the phases [33], which can indicate that Cr has been successfully doped into FeOOH to form FeCrO_3_. Surface morphology analyses were carried out on both FeOOH and FeCrO_3_/FeOOH, as shown in Figure 1b, short rod-shaped crystals are observed on the surface of FeOOH, whereas the surface of FeCrO_3_/FeOOH (Figure 1c) shows a regular hexahedral structure, and the size of the crystal size is also significantly increased. This suggests that Cr doping changes the surface morphology of the materials.

FeOOH and FeCrO_3_/FeOOH were activated at high temperature, and the two materials were transformed into Fe_2_O_3_ and FeCrO_3_/Fe_2_O_3_, respectively. Figure 1d shows the XRD patterns of Fe_2_O_3_ and FeCrO_3_/Fe_2_O_3_. The inability to identify the characteristic peaks of FeCrO_3_ alone may be due to the fact that the characteristic peaks of Fe_2_O_3_ are too strong, or due to the proximity of the characteristic peaks of FeCrO_3_ and Fe_2_O_3_. However, in FeCrO_3_/Fe_2_O_3_, the characteristic peaks corresponding to the (110) and (104) crystal faces of Fe_2_O_3_ are shifted to a lower angle, suggesting that the material was successfully doped with chromium. Compared with FeCrO_3_/Fe_2_O_3_, the diffraction peaks of pure Fe_2_O_3_ are wider (as shown in Figure 1e,f), which may be attributed to the larger grain size of FeCrO_3_/Fe_2_O_3_, resulting in the reduction in internal stresses inside the crystal, which leads to the narrowing of the diffraction peaks. In addition, the original morphological features of both Fe_2_O_3_ and FeCrO_3_ remain intact after the high-temperature activation treatment, indicating no signs of sintering or melting. This result indicates that the materials have good thermal stability and are suitable for thermal catalyst synthesis.

XRD and SEM information for the Fe_2_O_3_ and Cr/Fe_2_O_3_ materials used for the comparison of catalytic properties can be found in the SI (Figures S4 and S5). Based on Figure S4, it is evident that the characteristic peaks of Fe_2_O_3_ completely align with the standard card, signifying that the prepared Fe_2_O_3_ material is a pure phase. The Cr/Fe_2_O_3_ obtained by the impregnation method did not show the characteristic peaks of the Cr phase, which may be caused by the low content of Cr and its uniform dispersion on the surface of the material. Nonetheless, the characteristic peaks corresponding to the Fe_2_O_3_ (104) and (110) crystal planes shift towards lower angles, indicating the successful loading of Cr onto Fe_2_O_3_. It can be seen from Figure S5a–c that the Fe_2_O_3_ material has good crystallinity and uniform element distribution. Figure S5d shows that the Cr/Fe_2_O_3_ surface does not appear as a regular hexahedron like the FeCrO_3_/Fe_2_O_3_ (Figure 1f) material, which indicates that Cr only uniformly covers the short rod-like crystals on the surface of the material.

**Figure 2:**
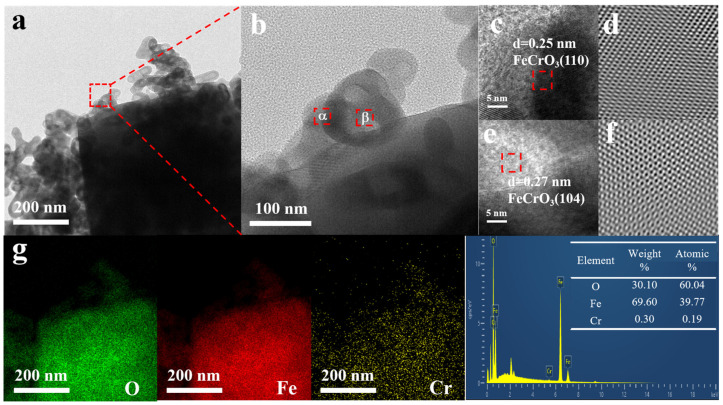
(**a**) TEM image, (**b**) HRTEM image, (**c**,**d**) IFFT image of the constituency α, (**e**,**f**) IFFT image of the constituency β, and (**g**) energy-dispersive X-ray spectroscopy (EDX) mapping images and spectra of FeCrO_3_/Fe_2_O_3_.

TEM analysis reveals the presence of hexahedral-shaped Fe-Cr crystals on the surface of the Fe matrix, as depicted in Figure 2a. The HPTEM images (Figure 2b) were Fourier transformed to determine two sets of lattice spacings of 0.25 nm and 0.27 nm, matching the (110) and (104) planes of FeCrO_3_. From Figure 2c,e, it can be seen that the crystallinity of FeCrO_3_/Fe_2_O_3_ is good. In addition, the EDX results (Figure 2g) indicate that the O, Fe, and Cr elements are uniformly distributed within the selected area of the nanocrystal.

**Figure 3:**
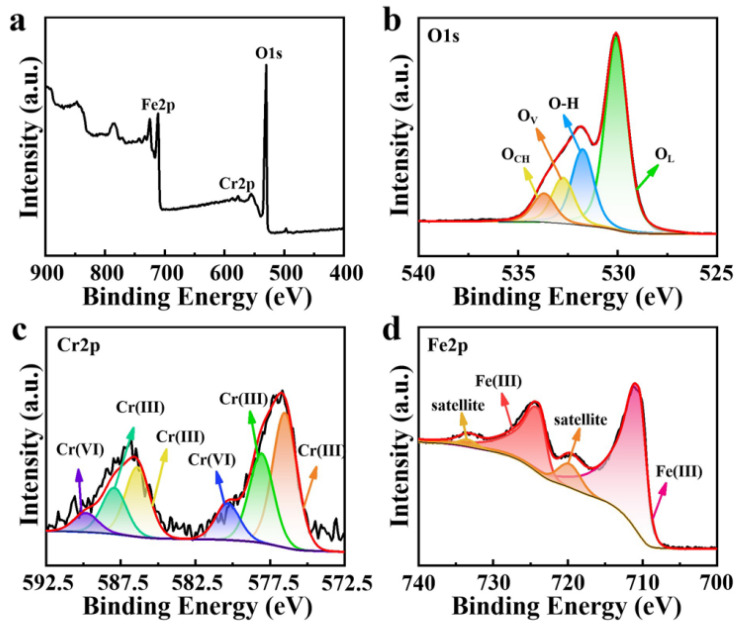
XPS spectra of FeCrO_3_/Fe_2_O_3_. (**a**) Full survey spectrum, (**b**) O 1s, (**c**) Cr 2p. and (**d**) Fe 2p. (The red line is the fitting curve obtained after the peak separation of XPS.)

The XPS spectrum of FeCrO_3_/Fe_2_O_3_ is shown in Figure 3a. Three peaks at 531 eV, 577 eV, and 711 eV, corresponding to the binding energies of O 1s, Cr 2p, and Fe 2p, respectively, indicate the presence of O, Cr, and Fe elements. The O 1s XPS spectra shown in Figure 3b indicate that the peak at 530 eV may be attributed to lattice oxygen, while the peaks at 531.7 eV, 532.7 eV, and 533.7 eV are attributed to O-H, oxygen vacancies, and chemisorbed oxygen [34,35]. As shown in Figure 3c, the Cr 2p XPS peaks observed at 586 eV and 577 eV correspond to the Cr 2p_1/2_ and Cr 2p_3/2_ peaks [36]. The peaks at 576.5 eV and 578.1 eV are attributed to Cr^3+^, which suggests that the valence state exists predominantly in the form of Cr^3+^ and that a small amount of Cr^6+^ may be the key to the better catalytic performance of the material in later stages. As shown in Figure 3d, the Fe 2p XPS peaks observed at 725.1 eV and 711.6 eV correspond to the Fe 2p_1/2_ and Fe 2p_3/2_ peaks [37,38,39], respectively. In conclusion, during the crystallization process, Fe and Cr are mainly present as Fe^3+^ and Cr^3+^, which is consistent with the elemental valence state of FeCrO_3_, and further proves that the combination of Cr and Fe forms stable FeCrO_3_ crystals instead of FeCr_2_O_4_ crystals [40]. The XPS spectra of the Fe_2_O_3_ and Cr/Fe_2_O_3_ catalysts are detailed in SI (Figures S6 and S7). In Figure S6b, it can be determined that Fe_2_O_3_ contains lattice oxygen, O-H, oxygen vacancies, and chemisorbed oxygen, and from Figure S6c, it can be determined that Fe_2_O_3_ contains only Fe^3+^. As shown in Figure S7, Cr/Fe_2_O_3_ contains lattice oxygen, O-H, and oxygen vacancies, and no Cr^6+^ peak is detected in this material, which may be an important reason for inhibiting its catalytic performance.

### 3.2. Formation Mechanism of FeCrO_3_

The chemical formula for iron titanium-type crystals is FeMO_3_ (M refers to metal elements), representing the substitution of a Fe atom in Fe_2_O_3_ with a metal atom M that possesses properties similar to Fe [41]. The proportion of metal cations in the crystals is influenced by factors such as the metal atoms’ radius, valence state, and gap size [42]. Consequently, substitution phenomena can occur among metal atoms with comparable properties under specific physical and chemical conditions. Common ilmenite-type crystals include FeTiO_3_, FeCrO_3_, FeGeO_3_, etc. [41,43,44]. Due to their similar atomic numbers (Fe-26 and Cr-24), Fe and Cr have analogous ionic radii and properties. Therefore, stable FeCrO_3_ crystals can form when appropriate molar ratios and hydrothermal conditions are present. Using a proper amount of ferric sulfate as a cocatalyst powder not only accelerates the formation of the ferrite matrix but also enhances the presence of free Cr^3+^, promotes its binding with FeOOH sites, and further accelerates the formation of FeCrO_3_.

**Figure 4:**
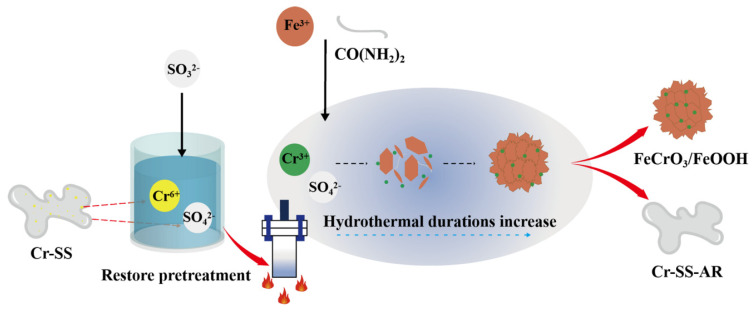
Schematic illustration of detoxification of Cr-SS and synthesis into FeCrO_3_/FeOOH.

In Figure 4, the detoxification mechanism of Cr(VI) from Cr-SS and its conversion into FeCrO_3_ is illustrated. The Cr-SS solution contains a high concentration of free Cr^6+^. Sodium sulfite (Na_2_SO_3_) provides SO_3_^2-^ during the reaction process, reducing free Cr^6+^ in chromic acid to Cr^3+^ (see reaction Formula (7)). During the hydrothermal stage, CO(NH_2_)_2_ is gradually hydrolyzed to provide an alkaline environment for the reaction (see reaction Formula (8)). Fe_2_(SO_4_)_3_, utilized as a cocatalyst powder, forms Fe(OH)_3_ under alkaline conditions (see reaction Formula (9)). As the hydrothermal reaction progresses, Fe(OH)_3_ begins to transform into FeOOH (see reaction Formula (10)). As shown in Figure S8, an iron matrix composed of Fe(OH)_3_ is initially formed, and FeOOH grows on its surface as the hydrothermal reaction continues. In an alkaline environment, free Cr^3+^ first forms Cr(OH)_3_ (see reaction Formula (11)). When Fe ions are abundant, under alkaline hydrothermal conditions, FeOOH and Cr(OH)_3_ are converted to the target product FeCrO_3_ at 180 °C (see reaction Formula (12)).

The chemical reaction formulas are described as follows:

Cr_2_O_7_^2−^ + 3SO_3_^2−^ + 8H^+^ → 3SO_4_^2−^ + 2Cr^3+^ + 4H_2_O (7)

CO(NH_2_)_2_ + 3H_2_O → CO_2_ + 2NH_4_^+^ + 2OH^−^ (8)

Fe^3+^ + 3NH_4_^+^ + 3OH^−^ → Fe(OH)_3_ + 3NH_4_^+^ (9)

(10) $$\mathrm{Fe}{\left(\mathrm{OH}\right)}_{3}\;\overset{180\;℃\;\mathrm{hydrothermal}}{↔}\;\mathrm{FeOOH}+H_{2}O$$

Cr^3+^ + 3NH_4_^+^ + 3OH^−^ → Cr(OH)_3_ + 3NH_4_^+^ (11)

(12) $$\mathrm{FeOOH}+\mathrm{Cr}{\left(\mathrm{OH}\right)}_{3}\overset{180\;℃\;\mathrm{hydrothermal}}{\to }{\mathrm{FeCrO}}_{3}+2H_{2}O$$

### 3.3. CO_2_ Hydrogenation Reaction Performance

**Figure 5:**
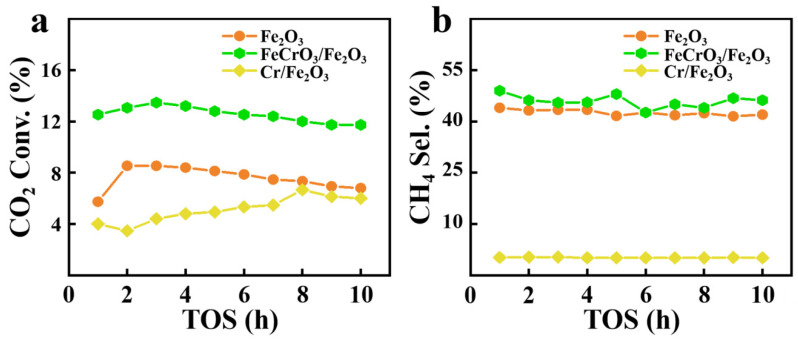
The (**a**) CO_2_ conversion rate and (**b**) CH_4_ selectivity rate of Fe_2_O_3_, FeCrO_3_/Fe_2_O_3_, and Cr/Fe_2_O_3_.

As shown in Figure 5a, FeCrO_3_/Fe_2_O_3_ exhibits higher CO_2_ conversion compared to Fe_2_O_3_ and Cr/Fe_2_O_3_. With the increase in reaction time, the CO_2_ conversion of FeCrO_3_/Fe_2_O_3_ did not change significantly, indicating its good stability. The CO_2_ conversion ability of Cr/Fe_2_O_3_ decreased in the first 2 h, then increased slowly, and reached the maximum at 8 h. This may be since Cr loaded by the impregnation method only deposits on the surface of the material, which inhibits the conversion of Fe_2_O_3_ to Fe-carbide and, thus, reduces the CO_2_ conversion. However, with the increase in reaction time, some chromium will be detached from the Fe_2_O_3_ surface, which may expose more iron sites, thus, improving the CO_2_ conversion.

As can be seen in Figure 5b, FeCrO_3_/Fe_2_O_3_ maintains a high CH_4_ selectivity throughout the reaction period, further highlighting its excellent stability. The results also show that Cr/Fe_2_O_3_ lacks selectivity for CH_4_, while Fe_2_O_3_ has more than 40% selectivity for CH_4_. This suggests that a single Cr species loaded on the surface of Fe_2_O_3_ has low selectivity for CH_4_ and even inhibits the original catalytic selectivity of the cocatalyst. As shown in Figure S8, the selectivity of FeCrO_3_/Fe_2_O_3_ for the C_2_–C_7_ product is twice that of Fe_2_O_3_. At the same time, the product selectivity of FeCrO_3_/Fe_2_O_3_ did not change significantly with the increase in reaction time, indicating that the material had good catalytic stability. The poor CO_2_ conversion and product selectivity of Cr/Fe_2_O_3_ may be due to the simple formation of CrOx particles rather than the formation of catalytically active chromium species. FeCrO_3_/Fe_2_O_3_ not only has a larger contact area but also prevents the cocatalyst from sintering by forming hexahedral crystals, which is conducive to the catalytic reaction. Meanwhile, we compared the performance of the catalysts prepared in this paper with that of the catalysts in the literature (see Table S3 for specific data) [27,45,46,47,48]. The results show that the low-temperature CO_2_ hydrogenation catalyst prepared by Cr-SS had better CH_4_ selectivity and certain CO_2_ conversion ability compared with the catalyst prepared by pure reagents.

### 3.4. Stability Evaluation

In order to better understand the mobility, stability, and toxicity of Cr in FeCrO_3_/Fe_2_O_3_ and Cr/Fe_2_O_3_ materials, an improved BCR method was used to determine the speciation of heavy metals in the samples, and toxicity risk assessment was conducted using the RAC method [49]. The content of different forms of Cr in FeCrO_3_/Fe_2_O_3_ and Cr/Fe_2_O_3_ and the RAC value of the material are detailed in SI (Table S4).

**Figure 6:**
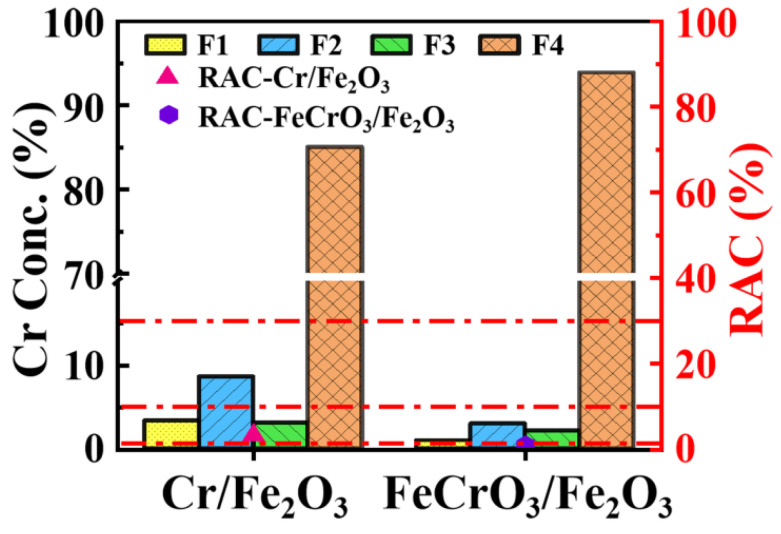
The different forms of Cr content and their RAC values in FeCrO_3_/Fe_2_O_3_ and Cr/Fe_2_O_3_. (The red dash line corresponds to RAC values of different scales.)

As shown in Figure 6, the values of F1 and F2 in FeCrO_3_/Fe_2_O_3_ are significantly lower than those in Cr/Fe_2_O_3_. The exchangeable metal form in the F1 part is usually considered the most easily transferable metal. Compared with FeCrO_3_/Fe_2_O_3_, Cr/Fe_2_O_3_ contains significantly higher Cr content in the exchangeable state, indicating a higher potential environmental risk. At the same time, it can be seen from the figure that the proportion of easily transitive forms (F1 and F2) of Cr in FeCrO_3_/Fe_2_O_3_ is relatively low, indicating that the directional conversion of Cr into FeCrO_3_ through hydrothermal pathways can better fix Cr, increase the proportion of stable state (F3 and F4), and effectively reduce the environmental risk level of the material [50]. At the same time, as shown in Table S5, the Cr^6+^ content of FeCrO_3_/Fe_2_O_3_ as an active site is very low, and the material exists in the form of iron–chromium crystals, indicating that it is not easy to be oxidized and the risk of poisoning is very low. Additionally, the RAC value of FeCrO_3_/Fe_2_O_3_ is below 1%, indicating it is a non-risk substance. In contrast, the RAC value of Cr/Fe_2_O_3_ is between 1–10%, and when the material is discarded and enters the natural environment, improper treatment can easily cause secondary environmental pollution.

## 4. Conclusions

In conclusion, this study introduces the direct conversion of Cr into FeCrO_3_/Fe_2_O_3_ through a straightforward hydrothermal reaction utilizing Cr-containing sodium sulfate (Cr-SS) as the raw material, followed by its application in low-temperature CO_2_ hydrogenation reactions. The incorporation of Cr prompts the transition of short rod-like crystals into hexahedral crystals, significantly increasing the crystal particle size. Moreover, the hydrothermal synthesis of FeCrO_3_ preserves the original O-H, oxygen vacancies, and adsorbed oxygen content of Fe_2_O_3_, ensuring the maintenance of its inherent catalytic performance. When employed in catalytic reactions, FeCrO_3_ achieves a CO_2_ conversion rate of 12.4% and a CH_4_ selectivity of 45.9%, surpassing the performance of Fe_2_O_3_ and Cr/Fe_2_O_3_ used as controls in the study. Furthermore, the proposed mechanism and corresponding equations elucidate the directional conversion of Fe^3+^ and Cr^3+^ into FeCrO_3_ during the hydrothermal process. Stability assessments conducted using the BCR and RAC methods confirm the risk-free nature of the FeCrO_3_ materials. This study not only accomplishes the removal and stabilization of chromium in Cr-SS but also yields a catalyst suitable for low-temperature CO_2_ hydrogenation reactions, presenting a valuable solution for the detoxification and recycling of chromium-containing wastes.

## Data Availability

Data are contained within the article and Supplementary Materials.

## Supplementary Materials

The following supporting information can be downloaded at: https://www.mdpi.com/article/10.3390/ma17071598/s1. Figure S1: Total Cr content in hydrothermal solution at different (a) reaction temperature, (b) reaction time, (c) dosage of Fe_2_(SO_4_)_3_ and (d) dosage of CO(NH_2_)_2_. Figure S2: (a)XRD pattern, (b) optical image of Cr-SS. (c) XRD pattern, (d) optical image of Cr-SS-AR. Figure S3: The overall morphology of (a–c) FeOOH and (d–f) FeCrO_3_/FeOOH. Figure S4: XRD pattern of Fe_2_O_3_ and Cr/Fe_2_O_3_. Figure S5: SEM-EDX images (a–c) of Fe_2_O_3_ and SEM-EDX spectra (d–f) of Cr/Fe_2_O_3_. Figure S6: XPS spectra of Fe_2_O_3_. (a) full survey spectrum, (b) O 1s and (c) Fe 2p. Figure S7: XPS spectra of Cr/Fe_2_O_3_. (a) full survey spectrum, (b) O 1s, (c) Cr 2p and (d) Fe 2p. Figure S8: SEM images of Cr-SS hydrothermal treatment for (a,b) 0.5 h, (c,d) 2 h, (e,f) 4 h, (g,h) 8 h and (i,j) 12 h. Figure S9: C_2_–C_7_ products selectivity of Fe_2_O_3_, FeCrO_3_/Fe_2_O_3_, and Cr/Fe_2_O_3_. Table S1: Cr content in the Cr-SS before and after treatment. Table S2: The comparison between the traditional and this paper in Cr removal from Cr-containing wastes. Table S3: The comparison of CO_2_ hydrogenation performance of different catalysts. Table S4: Different forms content and the RAC value of Cr in FeCrO_3_/Fe_2_O_3_ and Cr/Fe_2_O_3_. Table S5: Chromium content in different valence states for FeCrO_3_/Fe_2_O_3_ and Cr/Fe_2_O_3_.
