# Supplementary material for: Upcycling of Cr-Containing Sulfate Waste into Efficient FeCrO3/Fe2O3 Catalysts for CO2 Hydrogenation Reaction
Source: Materials (Basel). 2024 Mar 31;17(7):1598. doi: 10.3390/ma17071598 (PMC11012381; doi:10.3390/ma17071598)
Supplement: Supplementary file 1 [file materials-17-01598-s001.zip › materials-2925326-supplementary.pdf]

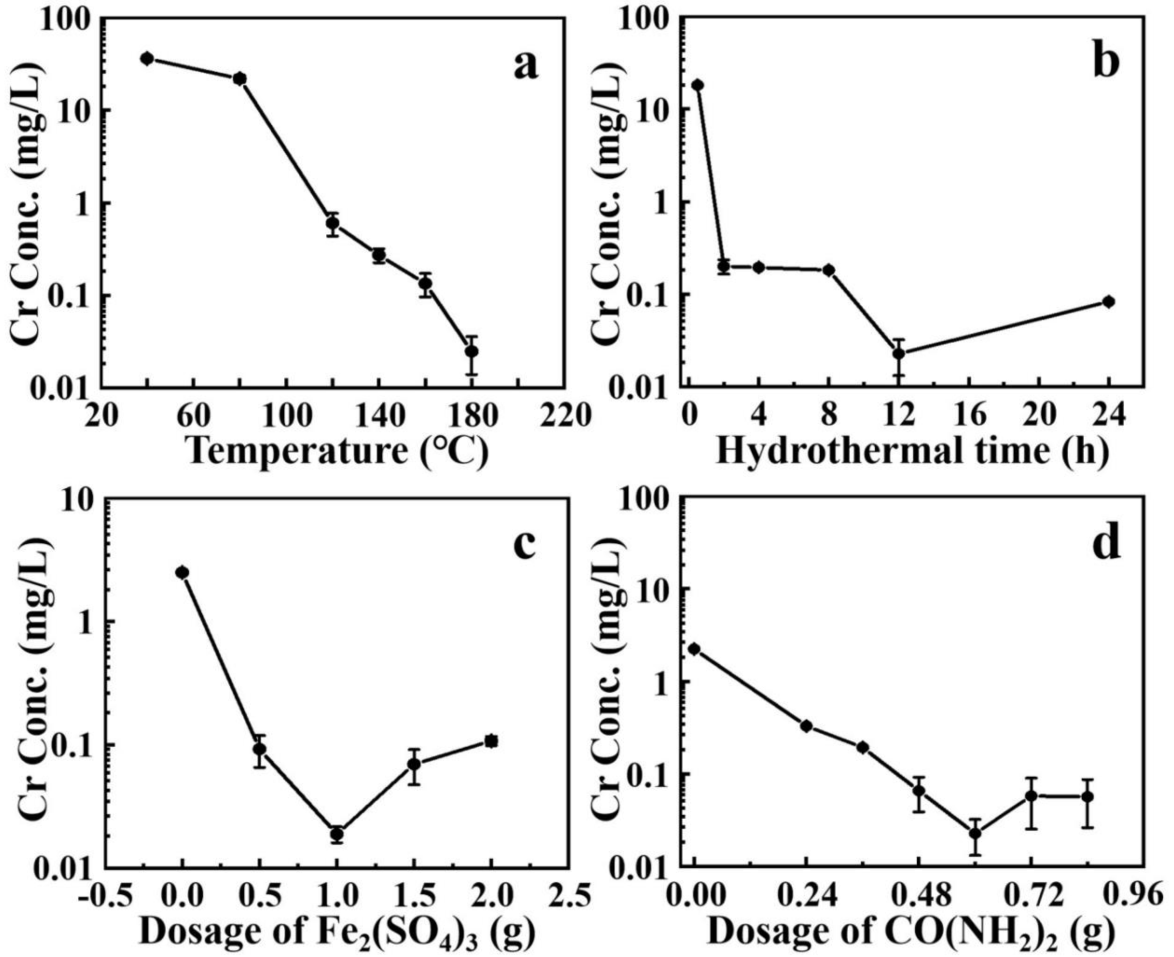

Fig. S1 Total Cr content in hydrothermal solution at different (a) reaction temperature, (b) reaction time, (c) dosage of $Fe_2(SO_4)_3$ and (d) dosage of $CO(NH_2)_2$.

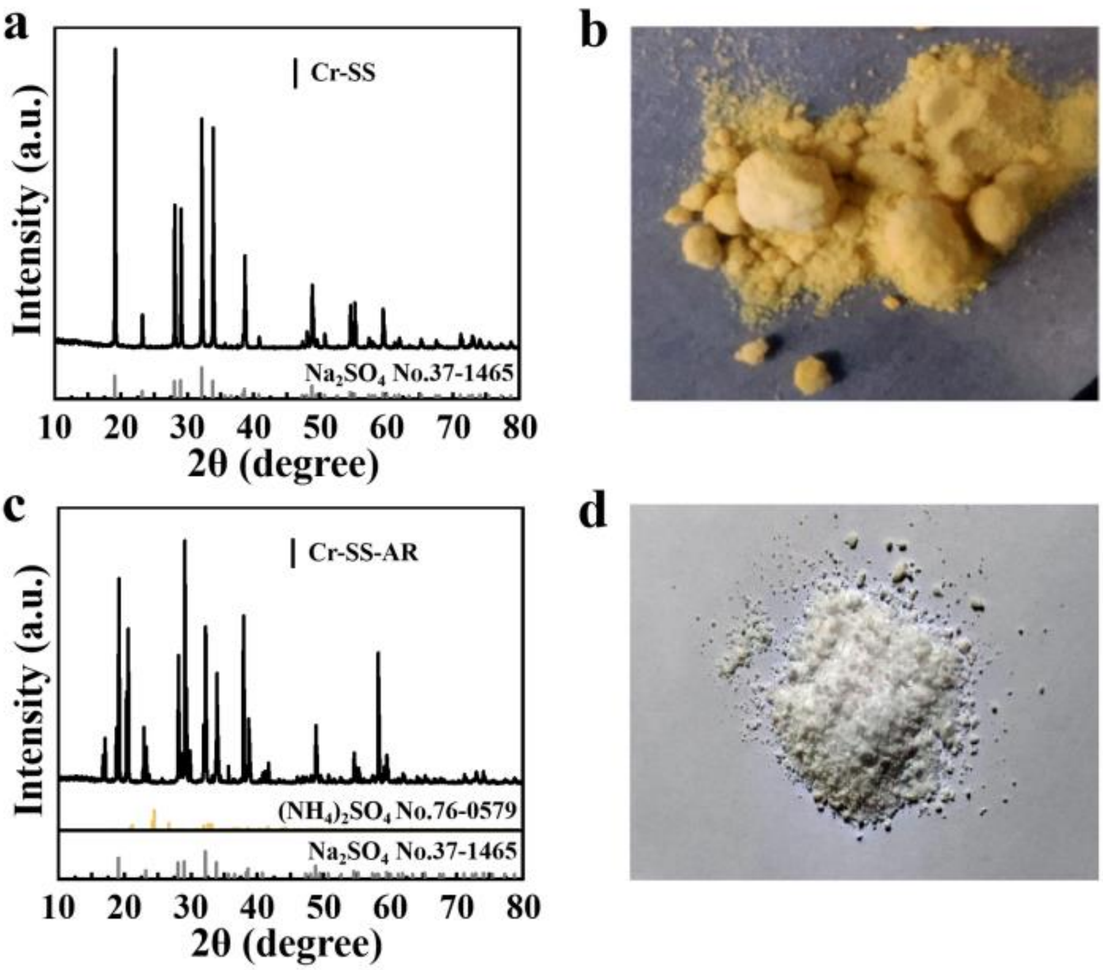

Fig. S2 (a)XRD pattern, (b) optical image of Cr-SS.

(c)XRD pattern, (d) optical image of Cr-SS-AR.

Table S1 Cr content in the Cr-SS before and after treatment.

Sample	Cr concentration (mg/L)	Fe concentration (mg/L)
Cr-SS	4.11958×10	0
Cr-SS-AR	1.27×10^{-2}	0

Table S2 The comparison between the traditional and this paper in Cr removal from Cr-containing wastes

Cr-containing wastes	Treatment method	Cr removal rate (%)	Reaction product	Reference
Chromium ore processing residue	$FeSO_4$ hydrothermal treatment	99.9	$FeCr_2O_4$	Lan et al., 2022 [1]
Chromium ore processing residue	$(NH_4)_2SO_4$ roasting H_2SO_4 leaching	95.39	$Na_2Cr_2O_7$	Zhang et al., 2022 [2]
Chromium ore processing residue	Chlorination roasting	99.9	$MgCr_2O_4$	Zhou et al., 2021 [3]
Cr-containing electroplating sludge	hydrothermal treatment	95	$FeCr_2O_4$	Xie et al., 2022 [4]
Chromium ore processing residue	glass-ceramic immobilization	77	$MgCr_{1.32}Fe_{0.19}$-$Al_{0.49}O_4$	Liao et al., 2017 [5]
**Cr-containing sulfate waste**	**hydrothermal treatment**	**99.9**	**$FeCrO_3$**	**This paper**

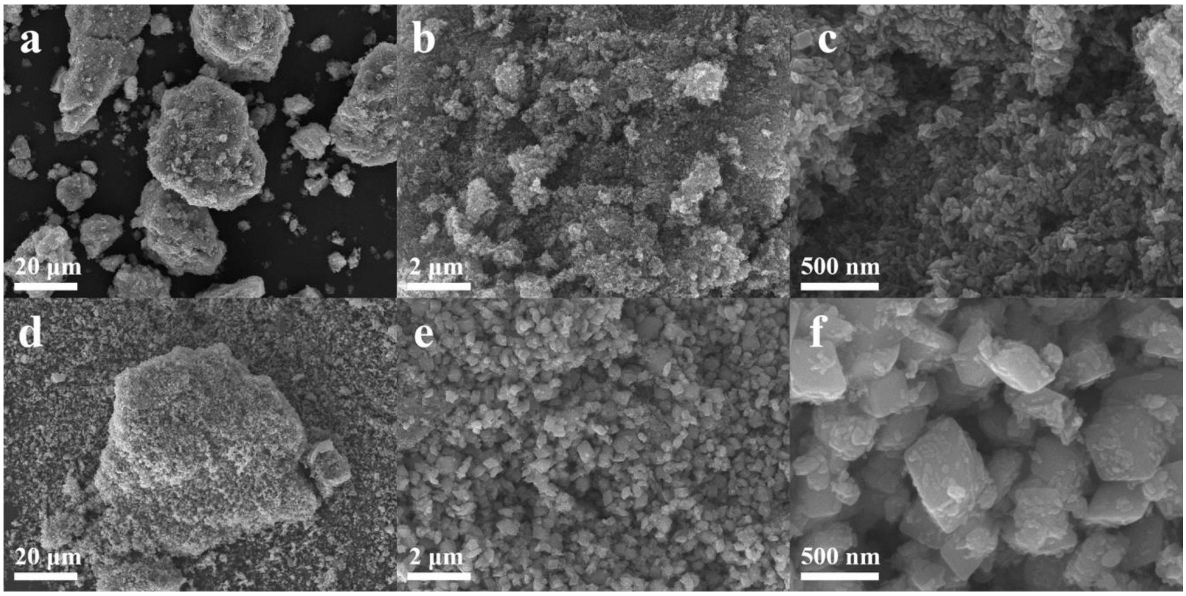

Fig. S3 The overall morphology of (a - c) FeOOH and (d - f) $FeCrO_3/FeOOH$.

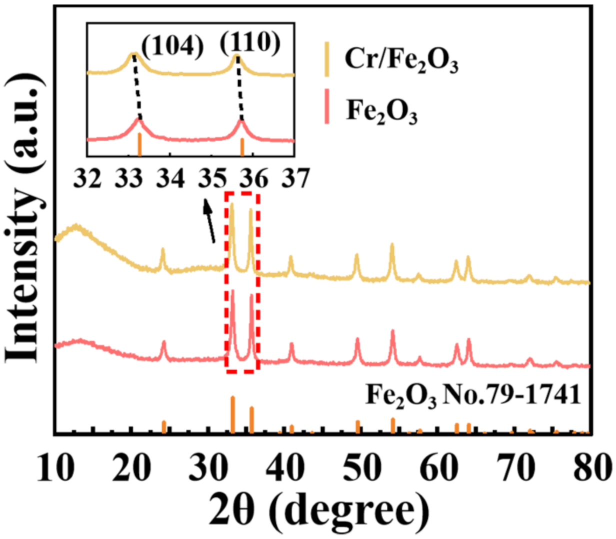

Fig. S4 XRD pattern of Fe_2O_3 and Cr/Fe_2O_3.

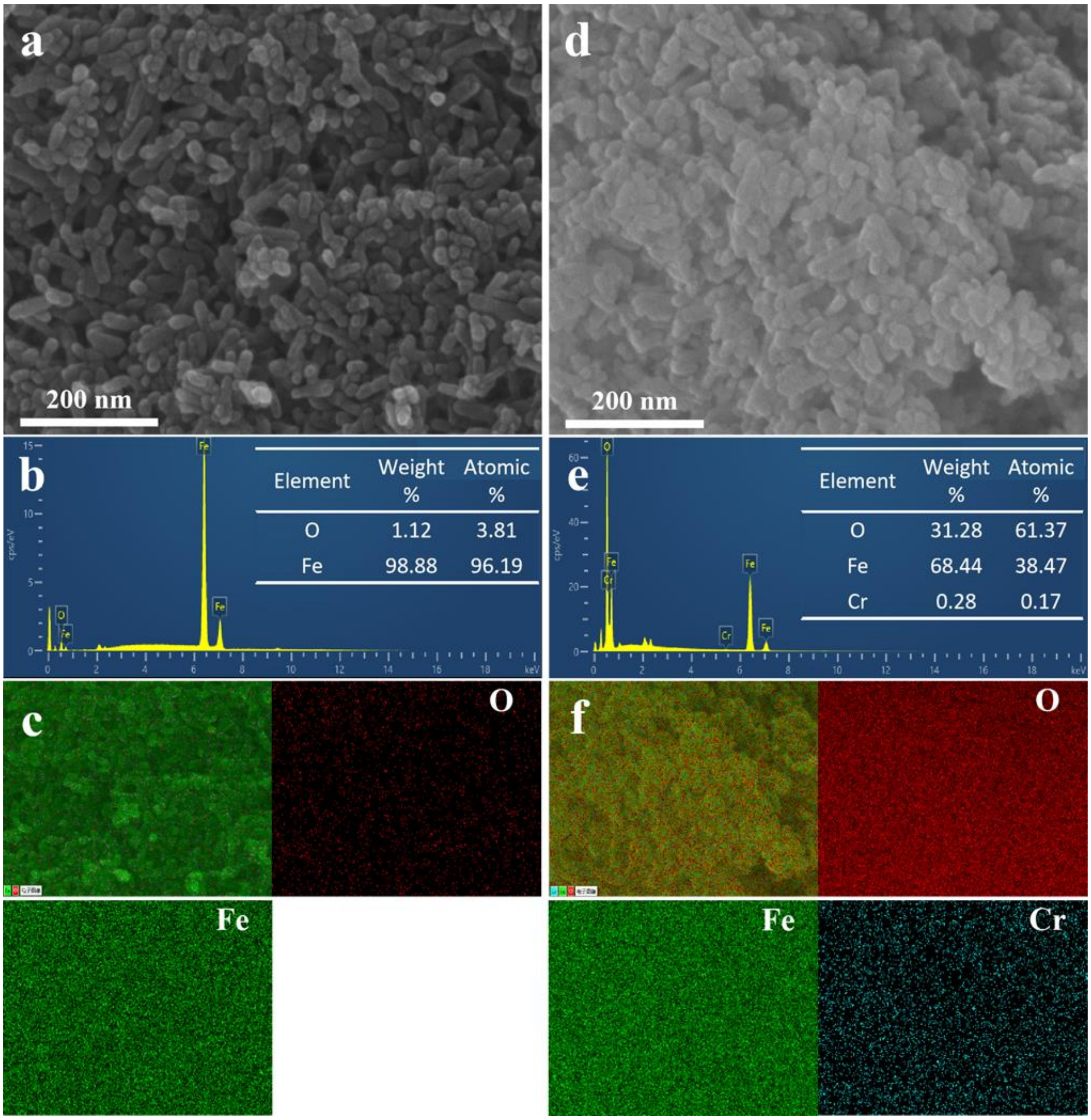

Fig. S5. SEM-EDX images (a, b, c) of Fe_2O_3 and SEM-EDX spectra (d, e, f) of Cr/Fe_2O_3.

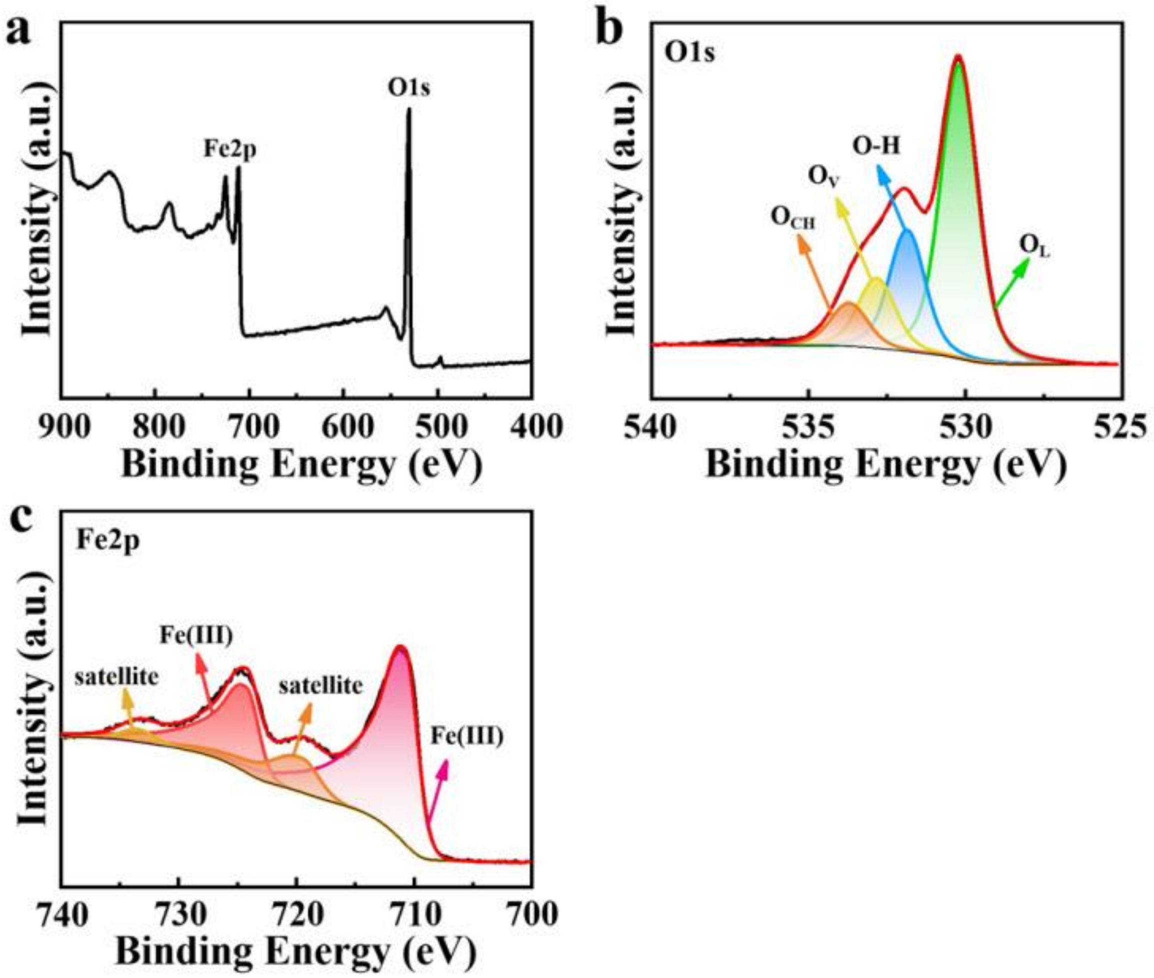

Fig. S6. XPS spectra of Fe_2O_3. (a) full survey spectrum, (b) O 1s and (c) Fe 2p.

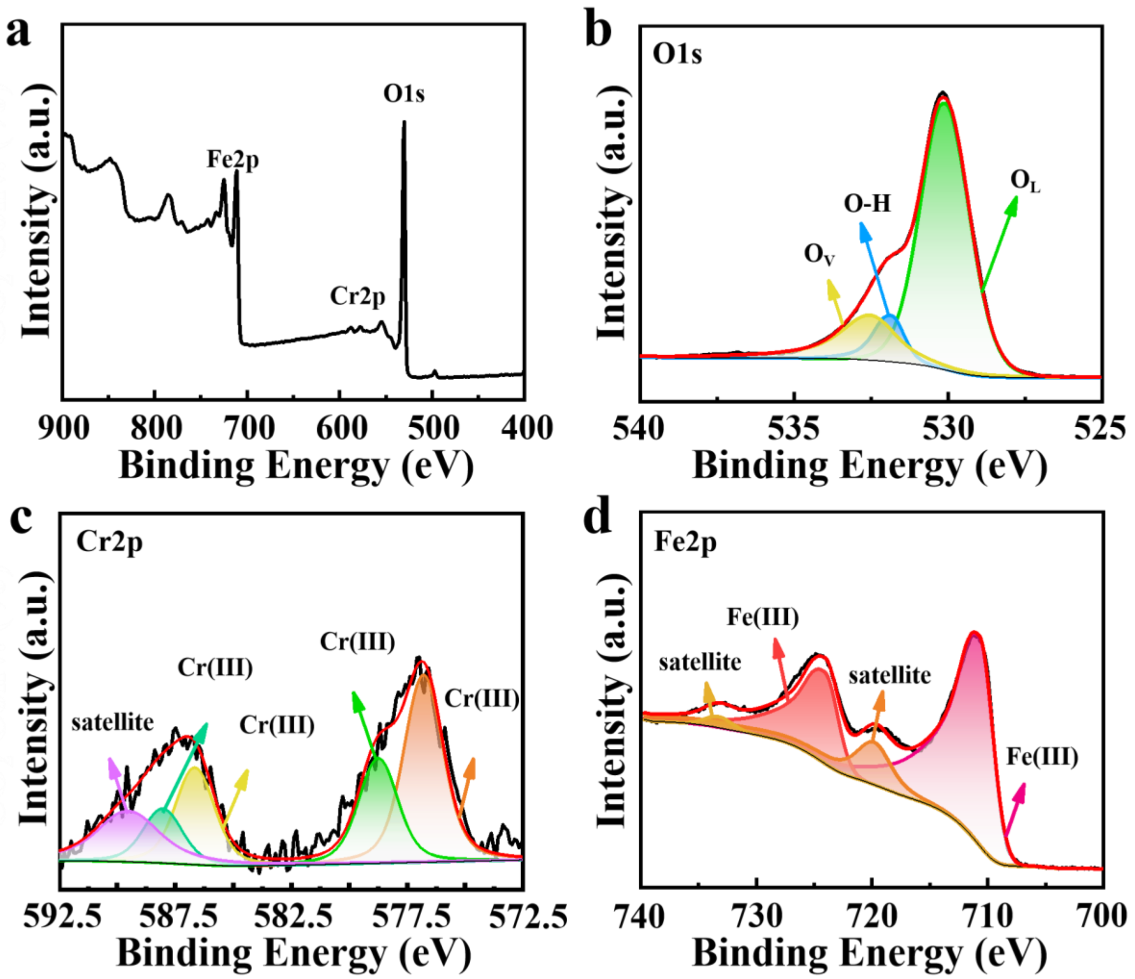

Fig. S7. XPS spectra of Cr/Fe_2O_3. (a) full survey spectrum, (b) O 1s, (c) Cr 2p and (d) Fe 2p.

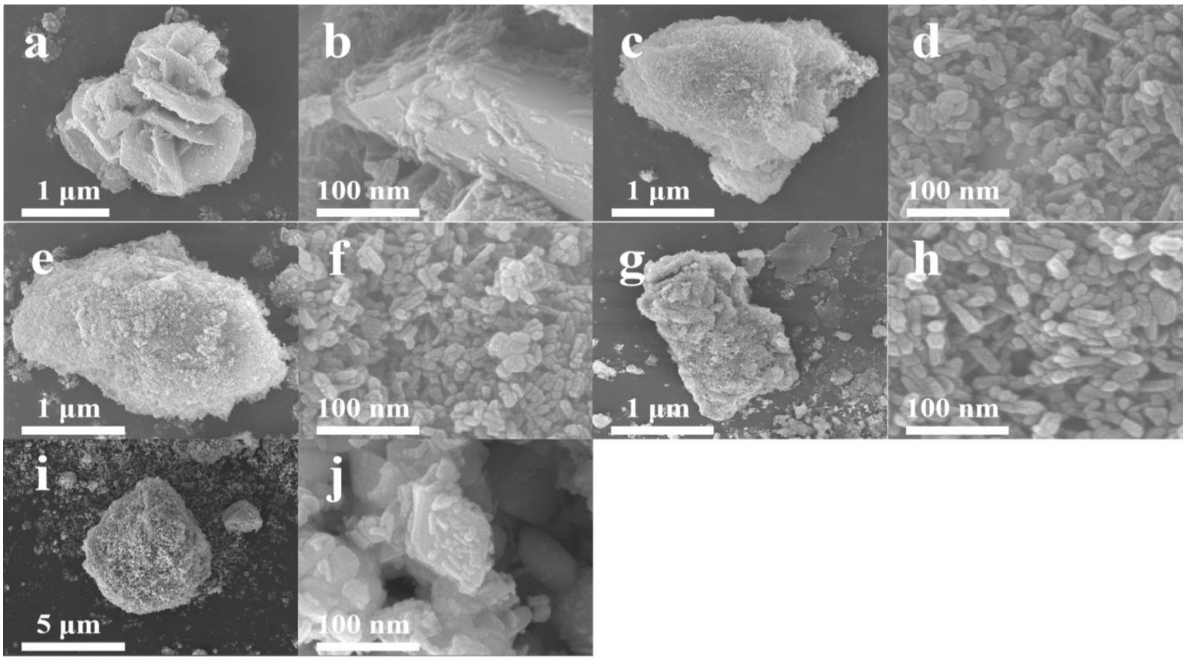

Fig. S8 SEM images of Cr-SS hydrothermal treatment for (a,b) 0.5 h, (c,d) 2 h, (e,f) 4 h, (g,h) 8 h and (i,j) 12 h.

During the hydrothermal reaction progression, Fe^{3+} undergoes conversion to $Fe(OH)_3$ in an

alkaline setting, serving as the precursor for the initial ferrite material formation. With prolonged hydrothermal time, the transformation of $Fe(OH)_3$ to FeOOH occurs, leading to the growth of FeOOH on the ferrite surface and the development of uniformly dispersed short rod-like crystals. After exceeding 8 h and reaching 12 h of hydrothermal treatment, the short rod-like crystals evolve into regular hexahedral crystals, resulting in a complete alteration of the ferrite surface morphology.

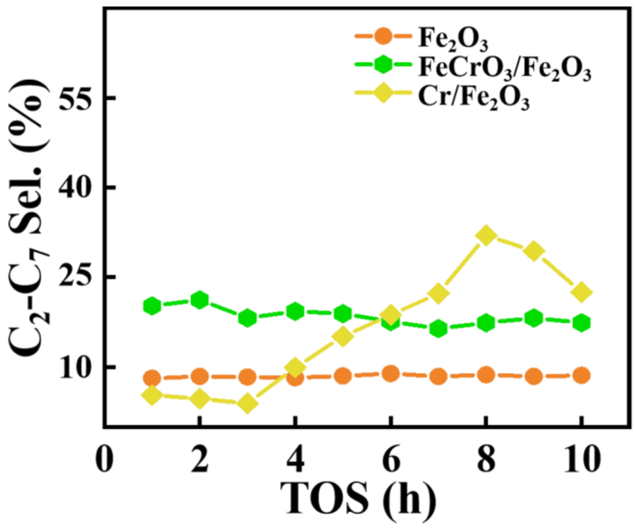

Fig. S9 C_2 - C_7 products selectivity of Fe_2O_3, $FeCrO_3/Fe_2O_3$, and Cr/Fe_2O_3.

Table S3. The comparison of CO_2 hydrogenation performance of different catalysts

Catalysts	Reaction temperature (°C)	CO_2 conversion (%)	CH_4 selective (%)	Selectivity of C_2-C_7 products (%)	Reference
FeZnK/ZrO_2	300	8.0	45.0	-	Pasupulety et al., 2024 [6]
CoCe-GC	600	23.4	37.4	-	Xie et al., 2023 [7]
2% Pt/ZrO_2	300	18	1.7	-	Seuser et al., 2023 [8]
1% Fe/13X	400	10	22	-	Franken et al., 2020 [9]
10% Ir/In_2O_3	200	17.7	-	70	Shen et al., 2021 [10]
Commercial Fe-Cr based catalyst	400	35	60	20	-
**$FeCrO_3/Fe_2O_3$**	**240**	**12.4**	**45.9**	**24.4**	**This paper**

Table S4 Different forms content and the RAC value of Cr in $FeCrO_3/Fe_2O_3$ and Cr/Fe_2O_3.

Sample	F1 (mg/kg)	F2 (mg/kg)	F3 (mg/kg)	F4 (mg/kg)	RAC value (%)
Cr/Fe_2O_3	2.27856×10^2	5.8158×10^2	2.10456×10^2	5.785625×10^3	3.348
$FeCrO_3/Fe_2O_3$	8.2688×10	2.49368×10^2	1.80324×10^2	7.85875×10^3	0.988

Table S5 Chromium content in different valence states for $FeCrO_3/Fe_2O_3$ and Cr/Fe_2O_3.

Sample	Cr^{3+} (mg/g)	Cr^{6+} (mg/g)
$FeCrO_3/Fe_2O_3$	1.03	1.2×10^{-2}
Cr/Fe_2O_3	1.08	-

Reference:

[1] Y. Lan, L. Zhang, X. Li, W. Liu, X. Su, Z. Lin, Efficient immobilization and utilization of chromite ore processing residue via hydrothermally constructing spinel phase $Fe^{2+}(Cr^{3+}_X, Fe^{3+}_{2-X})O_4$ and its magnetic separation, Sci. Total Environ. 813 (2022) 152637. https://dx.doi.org/10.1016/j.scitotenv.2021.152637

[2] J. Zhang, W. Xie, S. Chu, Z. Liu, Z. Wu, Y. Lan, V.V. Galvita, L. Zhang, X. Su, Sufficient extraction of Cr from chromium ore processing residue (COPR) by selective Mg removal, J. Hazard. Mater. 440 (2022) 129754. https://dx.doi.org/10.1016/j.jhazmat.2022.129754

[3] J. Zhou, X. Liu, J. Zheng, L. Li, W. Liu, L. Lin, Z. Lin, Simultaneous separation and immobilization of Cr(VI) from layered double hydroxide via reconstruction of the key phases, J. Hazard. Mater. 416 (2021) 125807. https://dx.doi.org/10.1016/j.jhazmat.2021.125807

[4] D. Xie, S. Chu, S. Zhang, A. Ivanets, L. Zhang, X. Su, Facile synthesis of Cr-doped ferrite catalyst from Cr-containing electroplating sludge with activated persulfate for efficient degradation of tetracycline, J. Environ. Chem. Eng. 10 (2022). https://dx.doi.org/10.1016/j.jece.2022.108805

[5] C.Z. Liao, Y. Tang, P.H. Lee, C. Liu, K. Shih, F. Li, Detoxification and immobilization of chromite ore processing residue in spinel-based glass-ceramic, J. Hazard. Mater. 321 (2017) 449-455. https://dx.doi.org/10.1016/j.jhazmat.2016.09.035

[6] N. Pasupulety, A.A. Alzahrani, M.A. Daous, H. Alhumade, CO_2-FT activity of Fe_7C_3 in FeZnK/ZrO_2 catalysts synthesized by using citric acid: Effect of pretreatment gas, Fuel. 360 (2024). https://dx.doi.org/10.1016/j.fuel.2023.130596

[7] H. Xie, N. Liu, J. Huang, S. Chen, G. Zhou, CoCe composite catalyst for the CH_4/CO_2 reforming reaction: Synergistic effects between Co and Ce species, J. Energy Inst. 111 (2023). https://dx.doi.org/10.1016/j.joei.2023.101389

[8] G. Seuser, M. Martinelli, E.S. Garcia, G.F. Upton, M. Ayala, J. Villarreal, Z. Rajabi, D.C. Cronauer, A.J. Kropf, G. Jacobs, Reverse water-gas shift: Na doping of m-ZrO_2 supported Pt for selectivity control, Appl. Catal., A 650 (2023). https://dx.doi.org/10.1016/j.apcata.2022.119000

[9] T. Franken, A. Heel, Are Fe based catalysts an upcoming alternative to Ni in CO_2 methanation at elevated pressure?, J. CO2 Util. 39 (2020). https://dx.doi.org/10.1016/j.jcou.2020.101175

[10] C. Shen, K. Sun, Z. Zhang, N. Rui, X. Jia, D. Mei, C.-j. Liu, Highly Active Ir/In_2O_3 Catalysts for Selective Hydrogenation of CO_2 to Methanol: Experimental and Theoretical Studies, ACS Catal. 11 (2021) 4036-4046. https://dx.doi.org/10.1021/acscatal.0c05628
